# Beyond quaternary ammonium compounds: Evaluating the harms of non-QAC disinfectant agents in healthcare and industry

**DOI:** 10.3205/dgkh000591

**Published:** 2025-11-07

**Authors:** Mitchell K. Ng, Michael A. Mont

**Affiliations:** 1 Department of Orthopaedic Surgery, Maimonides Medical Center, Brooklyn, NY, USA; 2 Department of Orthopaedic Surgery, Rubin Institute for Advanced Orthopedics, Baltimore, MD, USA

**Keywords:** disinfectants, non-quaternary ammonium compounds (QACs), healthcare risks, Ultraviolet-C (UV-C) disinfection

## Abstract

Quaternary ammonium compounds (QACs) account for approximately 35% of the U.S. disinfectant market, but mounting concerns about their links to occupational asthma, reproductive toxicity, and antimicrobial resistance as well as their allergic potential have driven increased adoption of non-QAC chemical disinfectants, which comprise the remaining 65% of the market.

This review critically examines the health, environmental, and material compatibility profiles of these non-QAC agents, including alcohols, chlorine compounds, hydrogen peroxide, peracetic acid, and phenolics. While many of these agents reduce certain risks associated with QACs, they present new challenges such as respiratory irritation, corrosiveness, systemic toxicity, and ecological persistence. Hydrogen peroxide and peracetic acid show favorable biodegradability but may impair wastewater systems or damage sensitive equipment. Among emerging technologies, ultraviolet-C (UV-C) disinfection stands out for its high antimicrobial efficacy, absence of chemical residues, and strong environmental profile. A risk-balanced, evidence-based approach to disinfectant selection is essential, and greater integration of UV-C and other non-chemical technologies may support safer and more sustainable infection control practices.

## Abstract (de)

Der Anteil quaternärer Ammoniumverbindungen (QAV) am US Desinfektionsmittelmarkt beträgt etwa 35%. Jedoch haben die zunehmende Besorgnis der Assoziation von QAVs mit berufsbedingtem Asthma, Reproduktionstoxizität und antimikrobieller Resistenz sowie die allergene Potenz zu verstärkter Einführung chemischer Desinfektionsmittel geführt, die keine QAVs enthalten und die derzeit die restlichen 65% des Markts ausmachen.

In der Übersicht werden die Gesundheits-, Umwelt- und Materialverträglichkeit nicht QAV basierter Desinfektionsmittel kritisch untersucht, darunter Alkohole, Chlorverbindungen, Wasserstoffperoxid, Peressigsäure und Phenole. Viele dieser Wirkstoffe verringern zwar bestimmte Risiken, die mit QAV verbunden sind, stellen aber auch neue Herausforderungen dar, wie z. B. Reizung der Atemwege, Korrosivität, systemische Toxizität und ökologische Persistenz. Wasserstoffperoxid und Peressigsäure sind zwar biologisch gut abbaubar, können aber Abwassersysteme beeinträchtigen oder empfindliche Geräte beschädigen. Unter den neuen Technologien zeichnet sich die UV-C-Desinfektion durch ihre hohe antimikrobielle Wirksamkeit, die Abwesenheit von chemischen Rückständen und ihr gutes Umweltprofil aus.

Ein risikoabgewogener, evidenzbasierter Ansatz bei der Auswahl von Desinfektionsmitteln ist von entscheidender Bedeutung, und eine stärkere Integration von UV-C und anderer nicht-chemischer Technologien kann sicherere und nachhaltigere Verfahren zur Infektionskontrolle unterstützen.

## Introduction

Chemical disinfectants are foundational to infection prevention across healthcare, food service, and industrial environments [1]. Their role in reducing microbial contamination on surfaces, instruments, and skin is critical to controlling healthcare-associated infections (HAIs), foodborne illness, and occupational exposure to pathogens. From routine surface cleaning to high-level sterilization of medical devices, chemical disinfectants enable rapid, scalable microbial control where heat or mechanical methods are impractical or insufficient [2].

Among the most widely used classes are quaternary ammonium compounds (QACs), valued for their broad-spectrum antimicrobial activity, ease of use, and material compatibility [3], [4]. They are frequently found in disinfectant wipes, sprays, and immersion solutions across clinical and commercial settings [5]. However, recent years have brought intensified scrutiny of QACs due to emerging evidence of their potential human health risks, including associations with occupational asthma, reproductive toxicity, and antimicrobial resistance [6]. These concerns have prompted regulatory bodies, health systems, and industry stakeholders to reassess the safety profiles of QACs and reconsider their widespread use – especially in settings involving frequent human exposure [7].

This shift has led to increased interest in non-QAC chemical disinfectants, including alcohols, chlorine compounds, hydrogen peroxide, peracetic acid, aldehydes, and phenolics [8]. These agents are often positioned as either safer, more environmentally friendly, or more effective alternatives to QACs – particularly in high-stakes environments like operating rooms, intensive care units, and food production facilities [9]. However, despite the growing reliance on these non-QAC agents, their adverse effects on human health, infrastructure, and the environment remain underappreciated and understudied [6]. REFERENCE 6 statt 10

This paper aims to critically evaluate the negative effects of non-QAC chemical disinfectants, drawing from toxicological, occupational, and environmental data. By highlighting their limitations and risks, this review seeks to inform safer disinfection strategies, guide product selection, and support policy shifts toward more sustainable infection control practices.

## Types of chemical disinfectants

By chemical class, while QACs make up 35% of the chemical disinfectant market [10], [11], [12], the remaining chemical disinfectants can be broadly classified into alcohols, chlorine-based compounds, hydrogen peroxide, peracetic acid, phenolics and other (aldehydes, other types of acids, etc.) (Table 1 (Tab. 1)).

## Alcohol-based disinfectants

Alcohols, such as ethanol and propan-2-ol, are ubiquitous in both medical and household disinfectant formulations [13]. They are commonly encountered in over-the-counter products like hand sanitizers, disinfecting sprays, and ethanol-based hand rubs (EBHR) and are often used at concentrations between 60% and 90% [14]. Their antimicrobial activity arises from their ability to disrupt lipid membranes and denature proteins, resulting in rapid microbial cell death [15]. Alcohols are particularly valued for their quick action, ease of application, and absence of chemical residues [14]. These features make them ideal for disinfecting small surfaces, skin, and delicate equipment. Nonetheless, their efficacy is significantly reduced in the presence of organic material, and they are ineffective against bacterial spores [16]. Additional drawbacks include their high flammability, potential for skin irritation or dryness, and the risk of inhalation-related symptoms. EBHR are essential for preventing infections caused by non-enveloped viruses. In opposite propanols are effective against enveloped viruses only, thus there are no other alternatives for virucidal hand antisepsis [17]. Long-term ingestion of ethanol in the form of alcoholic beverages can cause tumors. However, lifetime exposure to ethanol from occupational exposure < 500 ppm does not significantly contribute to the cancer risk. Mutagenic effects were observed only at doses within the toxic range in animal studies [17]. While reprotoxicity is linked with abuse of alcoholic beverages, there is no epidemiological evidence for this from EBHR use in healthcare facilities or from products containing ethanol in non-healthcare settings [17].

## Chlorine-releasing agents

Chlorine-based disinfectants – most notably sodium hypochlorite, commonly marketed as household bleach – are among the most widely used surface sanitizers in both institutional and residential settings in U.S. [18]. These agents exert their antimicrobial effect via oxidation of microbial proteins and nucleic acids, leading to widespread cell damage and death [19]. Chlorine compounds exhibit broad-spectrum activity against bacteria, viruses, fungi, and spores, and are relatively inexpensive and accessible [20]. Their typical applications include hospital surface decontamination, food preparation environments, and public sanitation efforts [19]. Despite their utility, chlorine compounds pose substantial health and environmental hazards. They are corrosive to many materials, particularly metals, and can generate toxic byproducts such as chloramines when mixed improperly [21]. Inhalation of chlorine vapors can result in respiratory distress, while direct contact may cause skin and eye irritation. Furthermore, chlorine-based disinfectants contribute to aquatic toxicity when released into wastewater streams [22].

## Hydrogen peroxide

Hydrogen peroxide is a reactive oxygen species frequently used for disinfection across healthcare, laboratory, and domestic settings [23]. Available in concentrations ranging from 3% (for consumer use) to 35% (for industrial sterilization), hydrogen peroxide is a broad-spectrum antimicrobial that inactivates pathogens by inducing oxidative stress – damaging DNA, proteins, and cellular membranes [23]. It is favored for its environmentally benign degradation into water and oxygen, which makes it attractive in settings prioritizing sustainability [24], [25]. In clinical environments, it is utilized in both liquid and vaporized hydrogen peroxide (VHP) forms for room and equipment decontamination. However, higher concentrations of hydrogen peroxide are associated with irritation to skin, eyes, and respiratory mucosa, and VHP use requires rigorous environmental controls to prevent occupational exposure [26]. Additionally, repeated exposure may lead to material degradation, particularly in rubber, silicone, and certain plastics.

## Peracetic acid

Acetic acid (PAA) is a potent oxidizing agent used in high-level disinfection [27], [28], particularly for heat-sensitive medical devices and in food manufacturing settings [29]. Commercially available as a mixture of acetic acid and hydrogen peroxide [30], PAA demonstrates exceptional efficacy against a wide array of pathogens, including bacterial spores, fungi, and mycobacteria [31]. Its oxidative mechanism targets protein sulfhydryl and disulfide bonds, disrupting cellular integrity and metabolism [32]. One of its major advantages lies in its biodegradability, breaking down into non-toxic end products like water, oxygen, and acetic acid, thus avoiding the formation of persistent environmental contaminants [32]. Nevertheless, its use is constrained by its high corrosiveness, especially toward metals and certain polymers, its significant respiratory toxicity [33] neurotoxic [34]. Symptoms similar to sick building syndrome are possible with daily large-area application. Exposure can provoke eye irritation, sore throat, coughing, and, in some cases, chemical pneumonitis among personnel working in inadequately ventilated areas [35]. The MAK value (maximum workplace concentration) for peracetic acid is 0.1 ppm (0.316 mg/m^3^) for short-term exposure (up to 15 minutes) and up to four times per shift; for longer exposures, an OEL (occupational exposure limit value) also applies, with 0.1 ppm; Because the odor threshold <0.15 mg/m^3^. The odor perception during surface disinfection is an alarm signal that the MAK value has been exceeded. Its sharp, vinegar-like odor also limits user acceptability in some settings [36].

## Phenolic disinfectants

Phenolic compounds constitute a class of disinfectants that includes agents such as 1,1'-Biphenyl]-2-ol (o-phenylphenol), 4-Chlor-3,5-dimethylphenol (chloroxylenol), and triclosan (TCS) [37]. Found in institutional cleaning products and some antiseptic formulations, phenolics function by disrupting microbial membranes and inhibiting key enzymatic pathways [37], [38]. They are relatively stable in the presence of organic matter and provide sustained antimicrobial activity, making them suitable for hospital surfaces, restroom sanitizers, and certain personal hygiene products [38]. However, phenolic compounds present several notable risks. They are capable of dermal absorption, and repeated exposure can lead to systemic toxicity, particularly in vulnerable populations such as infants and immunocompromised individuals [39]. Some phenolics, such as triclosan, have been banned or restricted due to concerns over endocrine disruption, antibiotic resistance promotion in vitro, environmental persistence [37]. Furthermore, phenolic disinfectants are acutely toxic to aquatic organisms, raising concerns about their widespread use and disposal. Human dermal absorption of triclosan (TCS) yields tissue levels similar to those linked with adverse effects in experimental models, including mitochondrial dysfunction [40]. TCS is minimally metabolized through the skin, with most of the compound remaining unchanged for at least 24 hours. Measurable biological effects can occur within hours of exposure [40]. TCS is also subject to both biological and chemical transformation, resulting in numerous by-products, many of which remain inadequately studied [40].

## Carbonic acids

Organic acids such as formic acid and fruit-derived acids like citric acid are increasingly included in environmentally labeled disinfectants due to their biodegradability, low systemic toxicity, and natural origin [41]. Formic acid, found in both fruit and insect venom, exhibits antimicrobial effects by lowering pH and disrupting microbial protein function [42]. It has shown utility in both agricultural and food-processing settings and is now gaining traction in healthcare applications.

Citric acid, already prevalent in consumer disinfecting wipes, acts by acidifying the cytosol and chelating metal ions, thereby impairing enzymatic activity and cell membrane stability [43]. These acids are generally well-tolerated and environmentally benign, breaking down readily without generating persistent toxic by-products [44].

However, their limitations include relatively weak antimicrobial potency, lack of residual activity, and reduced effectiveness in the presence of organic matter [45]. Formic acid, in particular, is volatile and may cause respiratory or skin irritation at high concentrations [42]. As such, while these compounds align with sustainability goals, their practical use is best suited to low-burden environments or as adjuncts in multi-agent formulations designed to reduce reliance on harsher disinfectants.

## Environmental impact of non-QAC disinfectants

While non-QAC disinfectants are frequently adopted as alternatives to compounds with known persistence and ecotoxicity, their environmental profiles are far from benign [2], [46]. The production, use, and disposal of these chemical agents can introduce a wide array of ecological risks, including aquatic toxicity, air pollution, and damage to wastewater systems [9]. As disinfection practices become more intensive in healthcare, food service, and industrial settings, understanding the downstream effects of these agents becomes increasingly important [19], [6].

Chlorine-based disinfectants, particularly sodium hypochlorite, are among the most problematic in terms of environmental harm [20]. Upon discharge into wastewater systems, chlorine reacts with organic matter to form halogenated byproducts, including trihalomethanes and haloacetic acids – some of which are carcinogenic, persistent, and toxic to aquatic organisms [22]. These compounds are difficult to remove during conventional wastewater treatment and can bioaccumulate in marine ecosystems. Additionally, improper mixing or disposal can result in the formation of toxic gases, increasing local air pollution and contributing to chemical hazards in enclosed environments [21].

Phenolic compounds also pose significant environmental concerns due to their poor biodegradability and high toxicity to aquatic life [47]. Phenols can persist in soil and sediment, where they disrupt microbial communities and hinder nutrient cycling. Even at low concentrations, compounds such as o-phenylphenol and chloroxylenol exhibit estrogenic activity and can interfere with endocrine systems in aquatic species [39]. Their inclusion in consumer and institutional cleaning products makes them a frequent contributor to municipal wastewater burdens.

Hydrogen peroxide, in contrast, is often cited for its environmental compatibility [24]. It degrades rapidly into water and oxygen, leaving no toxic residue, and does not produce harmful halogenated compounds. However, in concentrated forms or under specific conditions, hydrogen peroxide can contribute to increased chemical oxygen demand (COD) in effluent, potentially disrupting biological treatment processes in wastewater facilities [24]. Moreover, when used in vaporized forms (e.g., VHP), containment failures or improper handling can release aerosols into indoor air, where they pose risks to both human and environmental health [25], [48].

Peracetic acid shares many of hydrogen peroxide’s advantages in terms of breakdown products – it decomposes into acetic acid, water, and oxygen, all of which are considered environmentally benign [32]. Nonetheless, peracetic acid is highly reactive and contributes to COD surges in wastewater streams, which can negatively impact the microbial balance in treatment systems [31]. Additionally, its high oxidizing potential poses risks to non-target organisms if discharged in high volumes, particularly in food processing or agricultural runoff scenarios [36].

Alcohol-based disinfectants have a relatively low environmental footprint when used appropriately, but they are not without consequence [49]. Ethanol and propan-2-ol are volatile organic compounds (VOCs) that can contribute to tropospheric ozone formation, particularly when used in large volumes indoors or in poorly ventilated spaces [14]. While they readily biodegrade in aquatic systems, spills or overuse can increase localized biological oxygen demand, stressing small ecosystems [16].

Taken together, these findings challenge the assumption that non-QAC disinfectants are inherently “greener” or more sustainable [14]. While many of these compounds break down more easily than QACs and lack long-term environmental persistence, several still carry the risk of acute toxicity, byproduct formation, or system-level disruption. The ecological burden of disinfectants must therefore be weighed not only by their degradation rate, but by their total life cycle impact – from production and packaging to post-use environmental behavior. Regulatory frameworks and procurement policies should reflect this complexity by incorporating environmental safety as a key criterion in disinfectant selection.

## Discussion

As healthcare institutions, public health authorities, and industry stakeholders seek alternatives to QACs, non-QAC chemical disinfectants have gained prominence due to their established efficacy and, in some cases, favorable degradation profiles [14], [6]. However, the widespread assumption that these agents are inherently safer or more sustainable warrants careful re-examination [1], [2]. This review highlights that while non-QAC disinfectants address certain limitations of QACs – particularly in regard to antimicrobial resistance and environmental persistence – they introduce distinct and sometimes underrecognized health, environmental, and material hazards.

From a health and occupational safety standpoint, each non-QAC class presents a unique risk profile [14]. Propan-1-ol, while relatively benign in low exposure scenarios, can contribute to dermatitis [50]. Alcohols can impair the air quality issues in high-frequency use settings [49]. Chlorine compounds and aldehydes are well-established respiratory irritants and have been implicated in occupational asthma [22]. Peracetic acid, though increasingly used for high-level disinfection, is among the most concerning due to its acute airway toxicity [33]. The notion of substituting QACs with “greener” options like hydrogen peroxide and PAA must therefore be tempered by realistic assessments of risk [23], [48], particularly in healthcare environments where exposure can be frequent and intense.

Environmental considerations further complicate the narrative. While hydrogen peroxide and peracetic acid break down into biodegradable byproducts, others – such as chlorine and phenolic compounds – produce toxic or persistent residues that threaten aquatic life and compromise wastewater treatment infrastructure [23]. The unintended release of volatile organic compounds (VOCs) from alcohols also contributes to indoor air pollution and potentially to ozone formation [14]. These factors suggest that claims of environmental superiority must be substantiated not only through degradation rates but also through full life cycle analyses that account for production, use, disposal, and downstream effects.

In terms of infrastructure and material compatibility, non-QAC disinfectants pose several challenges. Chlorine-based agents and peracetic acid are corrosive to metals and polymers, while hydrogen peroxide vapor may damage electronics or compromise rubber seals [23]. These interactions can lead to premature equipment failure, elevated maintenance costs, and – even more concerning – safety risks to patients if critical instruments degrade unnoticed [51]. As hospitals increasingly rely on automated and digital equipment, material compatibility must be weighed more heavily in disinfectant selection.

The development of safer disinfectants will likely require multidisciplinary innovation, combining insights from microbiology, toxicology, materials science, and environmental engineering. Non-chemical disinfection modalities, including UV-C irradiation, ozone, and steam-based systems, also deserve greater exploration and investment, particularly in applications where chemical use is excessive or poorly tolerated [52], [53].

Finally, regulatory agencies and accrediting bodies play a vital role in standardizing labeling, exposure limits, and performance claims. Current guidance often fails to reflect the full scope of risk posed by widely used non-QAC disinfectants, leaving decision-makers without adequate tools for evidence-based selection.

In light of the limitations associated with both QAC and non-QAC chemical disinfectants, UV-C disinfection presents a compelling non-chemical modality that merits broader integration [53] (Table 2 (Tab. 2)). 

Across all major classes, chemical disinfectants suffer from several key drawbacks. Chief among these is the absence of visual feedback during use; users cannot see which surfaces have been adequately treated, leading to inconsistent coverage and missed areas. Similarly, there is no built-in method to confirm whether the correct dose has been applied or whether the required contact time has been achieved. Many of these agents pose health risks. From an environmental standpoint, many agents release volatile organic compounds (VOCs), contribute to aquatic toxicity, or strain wastewater treatment systems through increased chemical oxygen demand. Additionally, repeated or suboptimal use of any chemical disinfectant can foster microbial resistance, an emerging concern that parallels antibiotic resistance.

Taken together, these limitations underscore the need for disinfection solutions that are not only effective but also verifiable, safe, and environmentally sustainable. UV-C disinfection addresses many of these shortcomings by offering residue-free, precisely dosed, and visible coverage without introducing chemical hazards, positioning it as a next-generation alternative in both clinical and consumer settings. UV-C devices offer rapid, broad-spectrum inactivation of bacteria, enveloped viruses, and spores by disrupting microbial DNA and RNA through direct photolysis, without generating chemical residues or contributing to antimicrobial resistance [54], [55]. Because UV-C does not rely on liquid application, it eliminates many of the occupational hazards tied to volatile organic compounds, corrosive agents, and sensitizing chemicals. It also poses minimal environmental burden, as it produces no wastewater contaminants or persistent byproducts [56]. While limitations exist – most notably the need for direct line-of-sight exposure and the absence of residual antimicrobial activity – these can be mitigated by pairing UV-C with traditional agents in a layered or hybrid disinfection protocol. For reprocessing of medical devices, washer disinfectors for cleaning and disinfection are at present procedure of choice, but UV-C is in combination with pre-cleaning an alternative sustainable option. In high-risk environments such as operating rooms [53], ICUs, and cleanrooms, UV-C can serve as a valuable adjunct to manual disinfecting cleaning of surfaces, enhancing overall bioburden reduction while reducing reliance on harmful chemical loads. Its scalability, safety, and efficacy position it as a strategic asset in the pursuit of more sustainable and health-conscious infection control frameworks [53].

The Commission for Hospital Hygiene and Infection Prevention (KRINKO), under the authority of the Robert Koch Institute (RKI) in Germany [57], provides some of the most influential and widely cited infection prevention guidelines in Europe. KRINKO emphasizes evidence-based practices for disinfection and places particular importance on balancing antimicrobial efficacy with occupational and environmental safety [58]. KRINKO classifies disinfectants according to their intended use (e.g., surface, hand, instrument) and spectrum of activity, and recommends agents that are formally tested and listed by the VAH (German Association for Applied Hygiene) or demonstrate equivalence through independent data. Notably, KRINKO encourages limiting routine surface disinfection to high-risk areas – such as intensive care units, operating rooms, and infectious disease wards to reduce unnecessary chemical exposure and mitigate microbial resistance selection pressure [57]. With regard to non-QAC agents, KRINKO acknowledges the utility of alcohol-based disinfectants for hand hygiene and chlorine compounds, peracetic acid, and hydrogen peroxide for surface and instrument disinfection in appropriate contexts. However, the Commission highlights the need to avoid substances associated with significant health risks, particularly those with known allergenic or sensitizing potential [57]. For example, peracetic acid is recognized for its high-level efficacy but requires stringent control due to its respiratory toxicity and low occupational exposure limit (MAK value: 0.1 ppm). KRINKO guidance emphasizes adequate ventilation and worker protection measures when using such agents [28]. Importantly, KRINKO increasingly promotes non-chemical disinfection methods (e.g., UV-C irradiation) as viable adjuncts or alternatives, particularly in settings where chemical use poses occupational or environmental concerns. This aligns with broader trends in European infection control policy aimed at reducing chemical load, minimizing antimicrobial resistance, and enhancing workplace safety.

## Conclusion

This review highlights that while non-QAC chemical disinfectants offer viable alternatives to QACs, they are not without their own risks. Alcohols, chlorine-based agents, hydrogen peroxide, peracetic acid, and phenolics each present distinct concerns, including respiratory and skin toxicity, environmental persistence, and material incompatibility. These issues underscore the importance of evaluating disinfectants not just by efficacy but also by their occupational, ecological, and infrastructural impacts. Among emerging technologies, UV-C disinfection stands out for its high antimicrobial efficacy, chemical-free profile, and minimal environmental burden. Though it requires appropriate application and safety protocols, UV-C offers a promising path forward, particularly in environments seeking to reduce chemical exposure and improve sustainability.

Going forward, institutions should adopt a holistic, evidence-based approach to disinfectant selection – balancing effectiveness with human health and environmental stewardship. This will require cross-disciplinary collaboration, improved regulatory guidance, and openness to integrating non-chemical disinfection modalities into standard practice.

## Notes

### Authors’ ORCIDs:

- Mitchell K. Ng:
https://orcid.org/0000-0002-5831-055X
- Michael A. Mont:
https://orcid.org/0000-0003-4303-5556

### Funding

None. 

### Competing interests

MKN has received consulting fees from Stryker Inc., Johnson and Johnson Ethicon, Pacira BioSciences, Inc., Sage Products, Inc., Bonutti Technologies Inc., Hippocrates Opportunities Fund LLC, and Ferghana Partners, Inc. 

MAM has received consulting fees from 3M, Johnson & Johnson, Smith & Nephew, Kolon Tissuegene, Next Science, Pacira BioSciences, Inc., and Stryker; research funding from the National Institutes of Health, Kolon Tissuegene, and Stryker; is a shareholder for CERAS Health, Peerwell, and MirrorAR; serves as a board member for the Hip Society and the Knee Society, and is the Editor-in-chief for J Arthroplasty, and for J Knee Surg Surg Technol Internat Orthopaed. 

## Figures and Tables

**Table 1:**
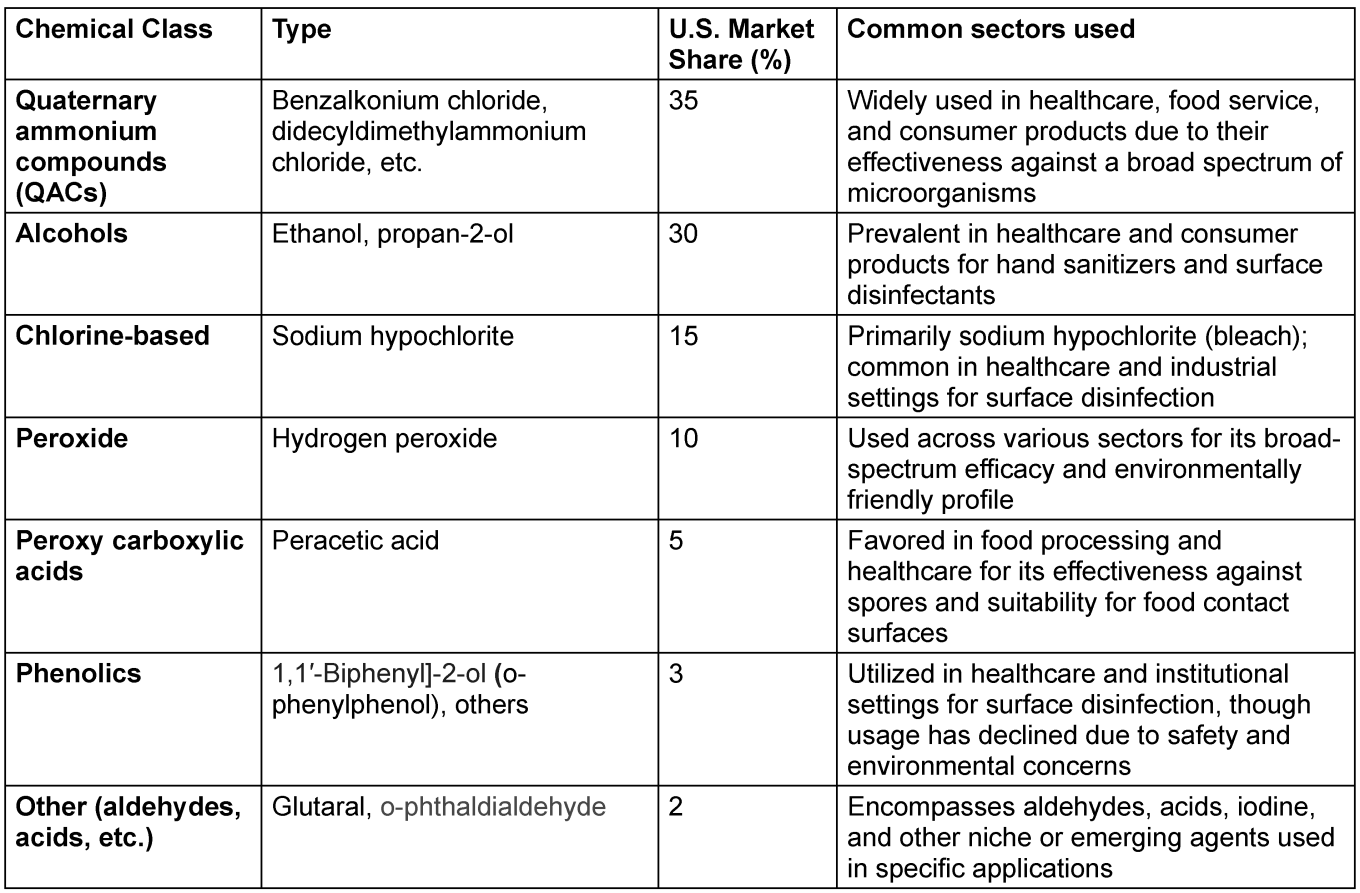
U.S. Disinfectant market by chemical class (2025 estimate)

**Table 2:**
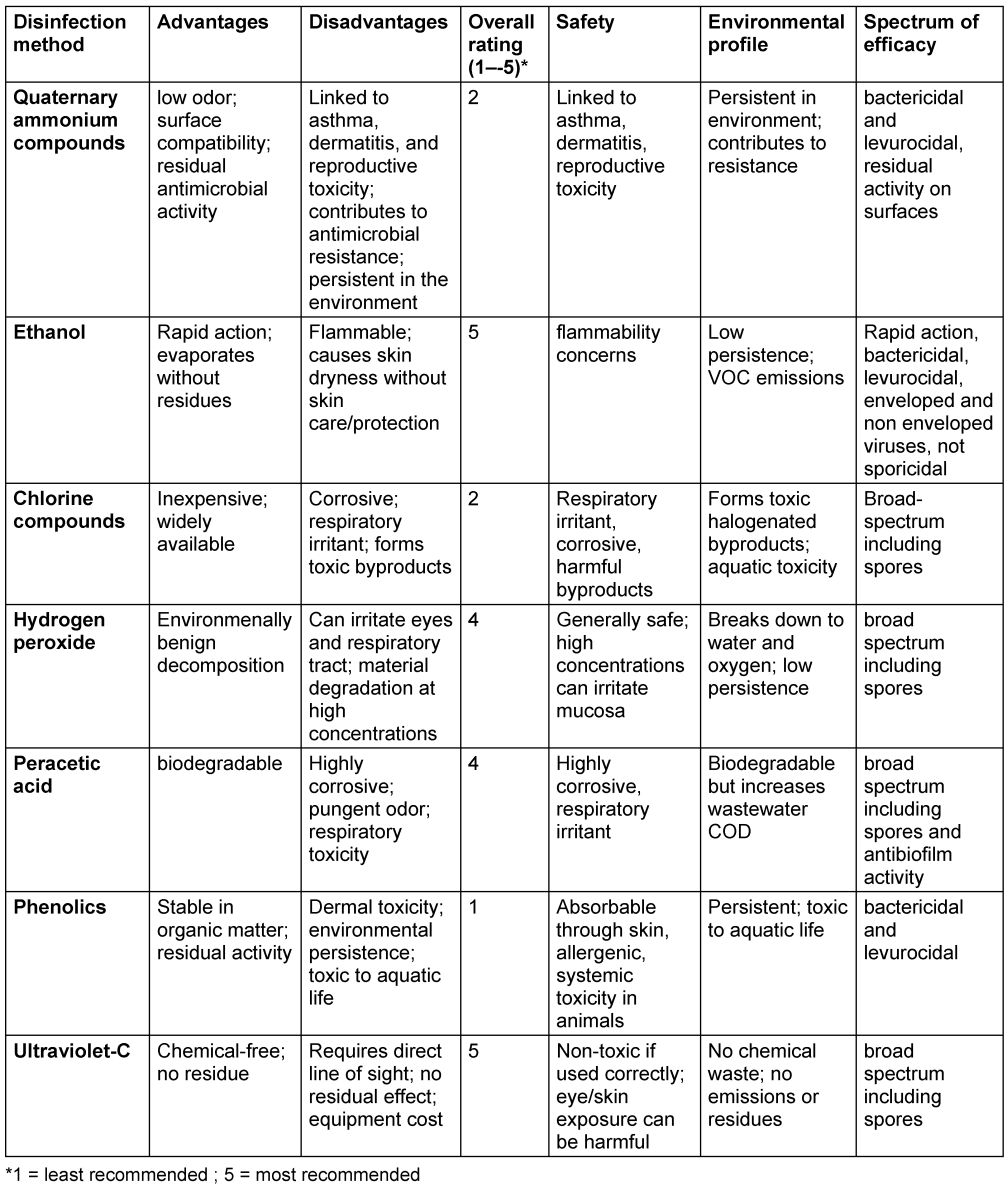
Risk-benefit assessment of disinfecting agents including UV-C
